# Nectar sugars and amino acids in day- and night-flowering *Nicotiana* species are more strongly shaped by pollinators’ preferences than organic acids and inorganic ions

**DOI:** 10.1371/journal.pone.0176865

**Published:** 2017-05-03

**Authors:** Kira Tiedge, Gertrud Lohaus

**Affiliations:** Molecular Plant Science/ Plant Biochemistry, University of Wuppertal, Wuppertal, Germany; Indian Institute of Science, INDIA

## Abstract

Floral nectar contains mainly sugars but also amino acids, organic acids, inorganic ions and secondary compounds to attract pollinators. The genus *Nicotiana* exhibits great diversity among species in floral morphology, flowering time, nectar compositions, and predominant pollinators. We studied nectar samples of 20 *Nicotiana* species, composed equally of day- and night-flowering plants and attracting different groups of pollinators (e.g. hummingbirds, moths or bats) to investigate whether sugars, amino acids, organic acids and inorganic ions are influenced by pollinator preferences. Glucose, fructose and sucrose were the only sugars found in the nectar of all examined species. Sugar concentration of the nectar of day-flowering species was 20% higher and amino acid concentration was 2-3-fold higher compared to the nectar of night-flowering species. The sucrose-to-hexose ratio was significantly higher in night-flowering species and the relative share of sucrose based on the total sugar correlated with the flower tube length in the nocturnal species. Flowers of different tobacco species contained varying volumes of nectar which led to about 150-fold higher amounts of total sugar per flower in bat- or sunbird-pollinated species than in bee-pollinated or autogamous species. This difference was even higher for total amino acids per flower (up to 1000-fold). As a consequence, some *Nicotiana* species invest large amounts of organic nitrogen for certain pollinators. Higher concentrations of inorganic ions, predominantly anions, were found in nectar of night-flowering species. Therefore, higher anion concentrations were also associated with pollinator types active at night. Malate, the main organic acid, was present in all nectar samples but the concentration was not correlated with pollinator type. In conclusion, statistical analyses revealed that pollinator types have a stronger effect on nectar composition than phylogenetic relations. In this context, nectar sugars and amino acids are more strongly correlated with the preferences of predominant pollinators than organic acids and inorganic ions.

## Introduction

Floral nectars are aqueous, carbohydrate-rich solutions that are secreted by nectaries of flowering plants. To a lower extent amino acids, organic acids, lipids, proteins, inorganic ions, scents and other secondary compounds are found in nectar [1–3], too. As an interface between the plant and its visitors, the nectar has at least two important functions: first, the attraction of specific pollinators that facilitate sexual reproduction and second, defense against nectar robbers, herbivores and pathogens [4–7].

Sugars dominate total nectar solutes and constitute the major energy source for visitors. The hexoses glucose and fructose as well as the disaccharide sucrose are highly abundant in nectar, although the ratio of sugars can differ interspecifically [1,8]. In some species there are also smaller amounts of other sugars present [9]. Nectar sugar concentration is higher in bee-pollinated flowers (35% (w/v)) than in flowers pollinated by butterflies or hummingbirds (20–25% (w/v)) [10], because the optimal nectar concentration is higher for viscous dippers than for suction feeders [11].

Besides sugars, nectar contains a wide range of different amino acids, which may primarily serve as a nitrogen source for the flowers’ visitors or as a phagostimulant [12]. More recent findings show highly variable concentrations of amino acids between different species and smaller variations in the composition of amino acids [13]. Pollinators’ preferences have the strongest influence on nectar composition, shown in a study about amino acid composition in Mediterranean floral nectars [14]. This impact is even more pronounced than environmental and taxonomical constraints. Among the measured amino acids, phenylalanine was most strongly correlated with pollinator preferences by having a phagostimulatory effect on several insects, especially on honey bees [14,15].

Organic acids like malic acid or citric acid play an important role in plant primary metabolism. Under physiological pH most of the organic acids are present in the anion form. Malate is the main soluble organic anion in several plant species and is, among others, a storage form for fixed carbon in leaves or other plant organs. Organic acids in nectars have not been studied in detail, despite the fact that they may play a role in nectar quality and pollinator attraction, e.g. by adding flavours to the nectar [16]. Apart from early studies demonstrating the presence of organic acids in various nectars [8,17], a more recent publication reported concentrations of organic acids in two *Aquilegia* species [16].

In addition to organic ions nectar contains several inorganic ions, whereby K^+^ is the dominant cation and Cl^-^ the dominant anion [18]. Ion concentration in nectar has a profound influence on the electrolyte balance of pollinators [19]. Furthermore, the ions could be part of the nectar redox cycle, a floral defense mechanism against microbial growth [7].

Several secondary compounds, which may mediate the specialization of plant-pollinator interaction, protect nectar from robbery or microbial activity and regulate the duration of pollinator visits, have also been identified in the nectar of some species [6,20,21]. For example, Kessler *et al*. [22] demonstrated that the occurrence of nicotine in *Nicotiana attenuata* significantly decreased both frequency and length of pollinator visits. The biological effects of secondary compounds are concentration dependent [6,20,21].

The pollination syndrome of a plant includes the floral morphology, the scent, and even the nectar composition, which are influenced by the preferences and needs of the pollinating animal [12]. Furthermore, shifts between pollinators has been one of the key explanations for the radiation of angiosperms [23,24]. Flowers pollinated by hummingbirds, Old World fruit bats, butterflies, moths, and long-tongued bees tend to secrete sucrose-rich nectar, whereas those pollinated by perching birds, New World bats, short-tongued bees, and flies tend to secrete hexose-rich nectar which may represent putative adaptations to dietary preferences of the respective pollinators [8]. But in other cases it appears that this model oversimplifies the complexity of floral evolution [25,26] and the influence of phylogenetic patterns was also shown [27]. Nevertheless, floral traits like the corolla tube length or flower opening time can limit the accessibility of nectar for a specific pollinator, e.g. if their mouthparts do not fit the requirements for the nectar intake [28].

So far there exist only a few studies that investigate differences of nectar compositions in day- and night-flowering plants. Jürgens (2004) analysed the sugar composition in nectar of diurnal and nocturnal *Conophytum* species (Aizoaceae) [29]. However, in some studies nectar sugar and/or amino acid compositions have been related to diurnal and nocturnal pollinators [30,31]. Since these pollinators visit either day- or night-flowering species those studies have also indirectly investigated the influence of flowering time on nectar composition. These investigations are usually limited to sugars and in some cases to amino acids.

The genus *Nicotiana* (Solanaceae) comprises 76 naturally occurring species including the important crop plant *N*. *tabacum *[32]. Goodspeed [33] classified the taxonomy, biogeography, and morphology present in this genus. Phylogenetic studies of the genus *Nicotiana* classified 13 sections: *Alatae*, *Nicotiana*, *Noctiflorae*, *Paniculatae*, *Petunoides*, *Polydicliae*, *Repandae*, *Rusticae*, *Suaveolentes*, *Sylvestres*, *Tomentosae*, *Trigonophyllae* and *Undulatae *[32,34]. Approximately 75% of *Nicotiana* species occur in South- and North-America and 25% in Australia; only one species has been found in Africa so far (*N*. *africana*) [33,35,36]. The greatest diversity of species can be found in the eastern Andes (South America), which led to the hypothesis that the genus evolved there and spread in a series of short and long distance moves to reach its current distribution [34].

As a first step towards investigating the role of pollinators in the evolution of nectar traits, Kaczorowski *et al*. [31] studied the specific *Nicotiana* section *Alatae*. In the present study the spectrum of species was expanded to 20 species from 11 sections. Species in the genus *Nicotiana* vary greatly in the time they flower (day versus night), floral morphology and in pollinator type with six different groups of pollinators visiting members of the genus. All different types of pollinators for tobacco plants (even the only bat-pollinated and the only sunbird-pollinated species) are covered. Therefore, the present study questions if nectar composition is influenced by pollinator types. Other constraints such as phylogenetic relations or ecological conditions could also have an impact on nectar composition. For this purpose, we investigated primary metabolites, which are involved in fundamental plant biochemistry processes (sugars, amino acids, organic acids), and inorganic ions in the nectar of related species with different pollinators. Such comprehensive studies about the occurrence of amino acids and organic acids among closely related species with different pollination types are rare, but they are necessary for a better understanding of the ecological role of these metabolites in nectar.

## Materials and methods

### Plant material

20 different species of the genus *Nicotiana* were examined. The seeds were provided by the University of Rostock (Germany), the Botanical Garden of the University of Bochum (Germany), and NiCoTa (Rheinstetten, Germany). Two sets (2014 and 2015) of at least three plants of every species were grown in a greenhouse. Each plant was potted in a single 5 L pot with compost soil. Cultivation was carried out with a 16-h-light/8-h-dark cycle, an irradiance of about 300 μmol photons m^-2^ s^-1^ and a temperature regime of 25°C day/18°C night. Corolla tube length and diameter were measured from six different, fully opened flowers per plant species and compared with already existing databases [31,33].

### Collection of nectar

All samples were collected on the first day of anthesis to minimize effects of flower aging on the nectar. Nectar samples were taken either with scaled micro-capillaries or with micropipettes for higher amounts. For longer flowers the corolla tubes had to be cut carefully to obtain access to the bottom of the calyx where the floral nectaries (nectar secreting glands) of *Nicotiana* species are located at the basal side of the gynoecium [18,37]. All samples were stored at -80°C until analysis. From each species 10 nectar samples (with the exception of *N*. *nudicaulis* with 8 samples) of different flowers from different plants were taken.

### Assay for microbial contamination

Yeasts or bacterial infections could alter the metabolite composition of nectar considerably by enzyme activity. To exclude microbial contamination, nectar samples of all plants were plated on malt extract and incubated for one week at 28°C.

### Collection of leaf samples and water:chloroform:methanol extraction

To verify that differences in nectar sugars were not due to differences in overall sugar content of the plants, sugar contents from leaf samples were analysed. From each species, 3 independent leaf samples from different individuals were collected. After shock freezing in liquid nitrogen, leaf tissue was extracted according to Nadwodnik and Lohaus [38].

### Analysis of sugars

The analysis of the nectar sugars via HPLC was performed according to Lohaus *et al*. [39]. An ion exchange column (CarbopacTM PA10 4x250mm; Dionex Corp, Sunnyvale, CA, USA) was eluted isocratically with 80 mM NaOH (JT Baker Chemicals). The sugars were detected by a pulse amperometric detector with gold electrode (ESA Model 5200, Coulochem II, Bedford MA, USA). Pulse setting was at 50, 700 and -800 mV for 400, 540 and 540 ms accordingly. For external calibration sugar standards (Sigma-Aldrich, Germany) were measured in parallel. The evaluation of the chromatograms was performed with an integration program (Peaknet version 5.1, Dionex).

### Analysis of free amino acids

The analysis of free amino acids was performed via HPLC according to Riens *et al*. [40] and Lohaus *et al*. [39]. For analysis of amino acids containing a primary amine group, precolumn derivatization with o-phtaldialdehyde was followed by the separation of the derivates on the reversed-phase column (Merck, Darmstadt, Germany) with an acetonitrile gradient. The derivates were detected by fluorescence. With this method, proline, an amino acid containing a secondary amine group, could not be detected. Therefore, for analysis of proline a precolumn derivatization with fluorenylmethyloxycarbonyl chloride (Sigma-Aldrich, Germany) instead of o-phtaldialdehyde was used. The derivates were detected by fluorescence (excitation 265 nm and emission 305 nm). For external calibration amino acid standards (Sigma-Aldrich, Germany) were measured in parallel. The evaluation of the chromatograms was performed with an integration program (Peaknet version 5.1, Dionex).

### Analysis of inorganic anions and organic acids

The analysis of anions and cations via HPLC was performed according to Lohaus *et al*. [41]. An anion exchange column (IonPacTM AS11 4x250mm; Dionex Corp, Sunnyvale, CA, USA) was eluted with a sodiumhydroxid gradient (4 to 77 mM in 30 min) for separation of the inorganic anions and organic acids. A suppressor was used to enhance the sensitivity by increasing the peak response and reducing background levels (ASRS Ultra II 4mm, Dionex USA). A cation exchange column (CS 12A, 4x250mm; Dionex Corp, Sunnyvale, CA, USA) with isocratic elution (20 mM H_2_SO_4_) was used for the separation of the cations. The ions were detected by their electronic conductivity (CP20 Conductivity Detector; Dionex USA). For external calibration standards were measured in parallel. The evaluation of the chromatograms was performed with an integration program (Peaknet version 5.1, Dionex).

### Statistical analysis

To determine if there is a significant difference between the groups of day- or night-flowering *Nicotiana* species a t-Test for independent samples (Student’s *t*-test) was applied. The normal distribution of the residuals was tested with Kolmogorov-Smirnov’s and the homoscedasticity (homogeneity of variances) was tested with Levene’s test. For samples that fit these requirements ANOVA was performed to analyse if the mean of one of the groups significantly distributes from the total mean of all samples. If this was the case, Tukey’s HSD was performed to analyse which group mean deviates from the others. For non-parametric data Independent-Samples Kruskal-Wallis test was performed for comparison of means between the groups. To examine whether nectar composition and floral traits were influenced by pollinator group or common ancestry, at first a Principal Component Analysis (PCA) was performed. All data were z-transformed before conducting the PCA to set their mean to 0 and the standard deviation (SD) to 1. Two principal components (PCs) were extracted from 33 independent initial variables, which represent the measurements of concentrations of all sugars, amino acids, inorganic anions, inorganic cations, and organic acids. Rotation Method was Varimax with Kaiser Normalization. Secondly a permutational multivariate analysis of variance (PERMANOVA) was conducted to identify the relative importance of the variables ‘Section’ and ‘Pollinator’ on the nectar composition. The *adonis* routine of the ‘vegan’ package in R was used for this purpose, which offers a multivariate analysis of variance using distance matrices based on permutation tests [42,43]. The Euclidean distance measure and 999 permutations were chosen to perform the PERMANOVA. All statistical analysis was performed by the use of IBM SPSS Statistics 22, except for the PCA and the PERMANOVA, which were performed using R (version 3.3.2, www.r-project.org).

## Results

### Nicotiana classification, pollinators and floral morphology

The classification of the *Nicotiana* species as day- or night-flowering was mainly based on information from the literature as well as on own observations (Table 1). The characteristics of night-flowering species were as follows: (i) flowers opening at night, (ii) corolla white to cream coloured and (iii) scent emission increase in the evening. The characteristics of day-flowering species were as follows: (i) flowers stay open at day and often also at night, (ii) corolla white, yellow, green or pink coloured and (iii) low scent intensity and no increase during nighttime. Ten species were classified as primarily day-flowering and ten species as night-flowering (Table 1). Corolla tube lengths of all examined tobacco species varied between 15 mm (*N*. *paniculata*) and 97 mm (*N*. *longiflora*) (Table 1).

**Table 1:** Overview of all examined *Nicotiana* species showing some of their main features.

Species	Section [32]	Origin [33]	Pollinator group	Flower colour	Corolla tube [mm]		Corolla length/ diameter	Corresponding picture in Fig 1
					length	diameter		
day-flowering								
*N*. *africana* Merxm.	Suaveolentes	AF	Nectariniidae [50]	yellow	32 ± 1	5.0 ± 0.1	6.4	i)
*N*. *attenuata* Torr. ex Wat.	Petunoides	NA	Trochilidae [54]	white	27 ± 1	3.2 ± 0.1	9.0	g)
*N*. *glauca* Graham	Noctiflorae	SA	Trochilidae [47]	yellow	33 ± 3	4.5 ± 0.2	7.3	k)
*N*. *knightiana* Goodsp.	Paniculatae	SA	Trochilidae^a^	yellow	24 ± 1	4.0 ± 0.1	6.0	f)
*N*. *langsdorffii* Weinm.	Alatae	SA	Trochilidae [25]	yellow	21 ± 2	4.7 ± 0.2	4.3	e)
*N*. *nudicaulis* Watson	Repandae	SA	Apidae [52]	white	17 ± 0	3.2 ± 0.1	5.7	b)
*N*. *palmeri* Gray	Trigonophyllae	NA	Trochilidae [48]	white	21 ± 1	3.8 ± 0.2	5.5	d)
*N*. *paniculata* L.	Paniculatae	SA	Trochilidae [26]	white	15 ± 1	4.6 ± 0.2	3.3	a)
*N*. *rustica* L.	Rustica	NA	Apidae [25]	yellow	18 ± 0	6.9 ± 0.9	2.6	c)
*N*. *tabacum* L.	Nicotiana	SA	Trochilidae^a^	pink	43 ± 2	7.0 ± 0.2	6.1	m)
night-flowering								
*N*. *acuminata* Hook.	Petunoides	SA	Sphingidae [32]	white	75 ± 1	3.0 ± 0.5	25.0	p)
*N*. *alata* Link & Otto	Alatae	SA	Sphingidae [45]	white	87 ± 5	3.5 ± 0.5	24.9	s)
*N*. *benthamiana* Domin	Suaveolentes	OC	self pollination [55]	white	31 ± 1	2.0 ± 0.3	15.5	h)
*N*. *longiflora* Cav.	Alatae	SA	Sphingidae [49]	white	97 ± 4	2.6 ± 0.1	38.8	t)
*N*. *nesophila* Johnston	Repandae	SA	Sphingidae [52]	white	43 ± 2	1.5 ± 0.1	28.7	o)
*N*. *otophora* Griseb.	Tomentosae	SA	Glossophaginae [44]	yellow	33 ± 1	5.0 ± 0.3	6.6	j)
*N*. *plumbaginifolia* Viv.	Alatae	SA	self pollination [31]	white	40 ± 2	2.0 ± 0.1	20.0	l)
*N*. *stocktonii* Brandegee	Repandae	NA	Sphingidae [52]	white	61 ± 2	2.5 ± 0.1	24.4	q)
*N*. *suaveolens* Lehm.	Suaveolentes	OC	Sphingidae [25]	white	42 ± 1	3.1 ± 0.2	14.0	n)
*N*. *sylvestris* Speg. & Com.	Sylvestres	SA	Sphingidae [52]	white	75 ± 8	3.0 ± 0.1	25.0	r)

^a^classified via flower morphology/ pollination syndrome.

Abbreviation: AF = Africa, OC = Australia, NA = North America, SA = South America.

**Fig 1:**
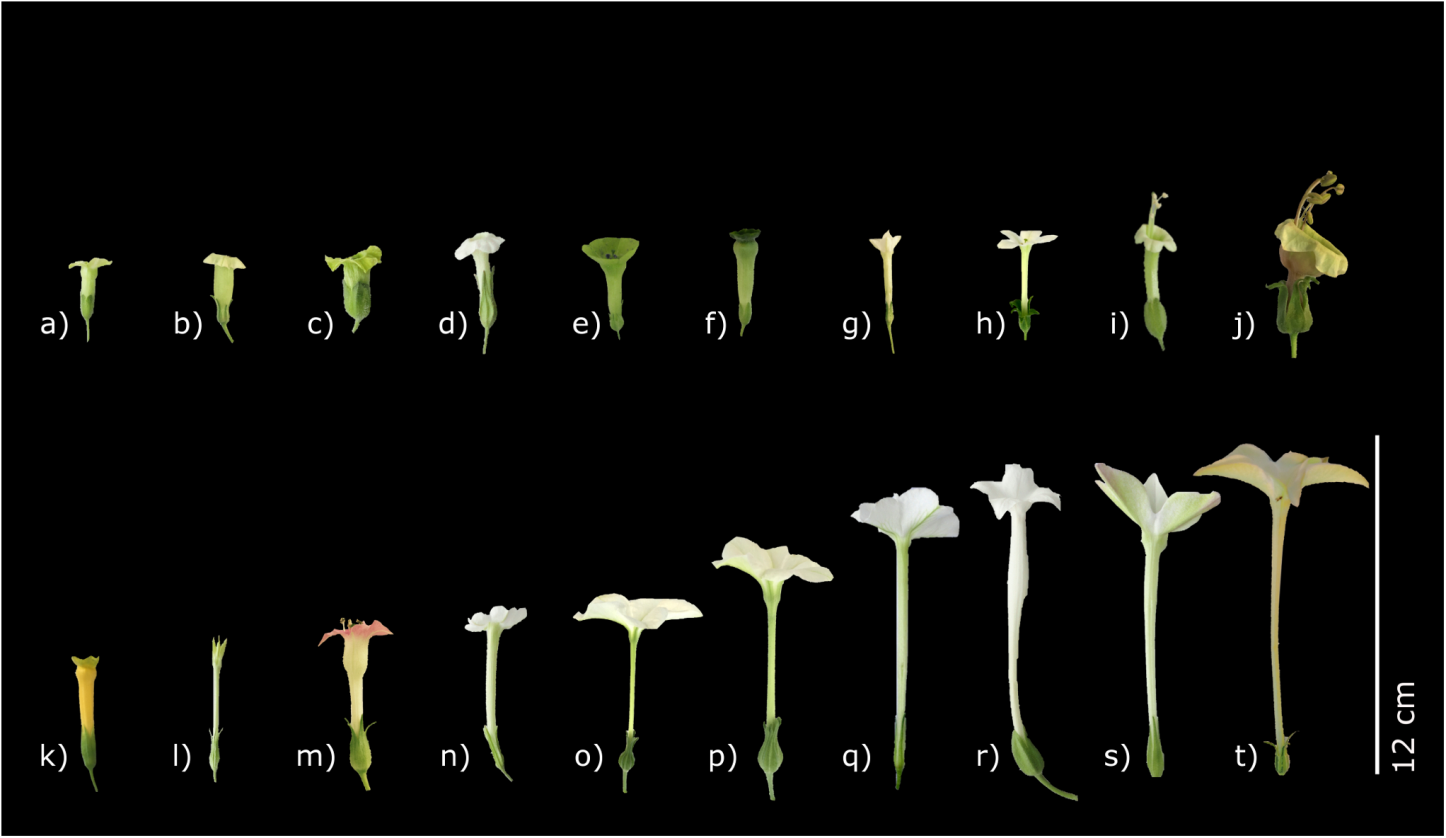
Flowers of the examined *Nicotiana* species. a) *N*. *paniculata* b) *N*. *nudicaulis* c) *N*. *rustica* d) *N*. *palmeri* e) *N*. *langsdorffii* f) *N*. *knightiana* g) *N*. *attenuata* h) *N*. *benthamiana* i) *N*. *africana* j) *N*. *otophora* k) *N*. *glauca* l) *N*. *plumbaginifolia* m) *N*. *tabacum* n) *N*. *suaveolens* o) *N*. *nesophila* p) *N*. *acuminata* q) *N*. *stocktonii* r) *N*. *sylvestris* s) *N*. *alata* t) *N*. *longiflora*

The colour (white, greenish or rosy pink) and the flower morphology of the species are shown in Fig 1. All of the *Nicotiana* flowers in the upper row of Fig 1 have comparatively short corolla tubes (< 40 mm) and belong to day-flowering species with the exception of h) *N*. *benthamiana* and j) *N*. *otophora* (night-flowering species). The lower row represents species with longer corolla tube length (> 40 mm) exhibiting nocturnal anthesis with the exception of k) *N*. *glauca* and m) *N*. *tabacum*.

The classification of *Nicotiana* species in pollination types and information on flower visitors are shown in Table 1. In their natural environment species with longer, more slender floral tubes and white flowers are pollinated by hawk moths (Sphingidae) as their main nocturnal visitors, and short-tubed, coloured flowers are mainly pollinated by hummingbirds (Trochilidae) [26,31,44–49]. The nocturnal species *N*. *otophora* is pollinated by nectar feeding bats (Glossophaginae) [44]. In the case of *N*. *africana* sunbirds (Nectariniidae) are the predominant pollinators and *N*. *nudicaulis* and *N*. *rustica* are basically visited by bees (Apidae) [25,50,51,52]. *N*. *attenuata* is pollinated by both nocturnal hawk moths and diurnal hummingbirds [53,54]. Because *N*. *attenuata* shows several floral characters which are more attractive to hummingbirds [22,54], this species is grouped with the day-flowering species (Table 1). *N*. *plumbaginifolia* and *N*. *benthamiana* are both described as autogamous [31,55] whereas no flower opening was observed at all for *N*. *plumbaginifolia*. Still *N*. *plumbaginifolia* may be classified as nocturnal due to its increased nectar secretion at night.

### Concentrations of sugars in nectar

Nectar of all 20 *Nicotiana* species contained the three main sugars glucose, fructose and sucrose (Table 2). No other mono-, di- or oligosaccharides were detected in appreciable quantities. The total sugar concentrations ranged from 417 mM (10.9% (w/v)) in *N*. *nesophila* to 1984 mM (42.7% (w/v)) in *N*. *nudicaulis*. The highest sucrose-to-hexose ratio of 2.0 was found in the night-flowering *N*. *nesophila*, the lowest ratios (0.1) were found in the day-flowering species *N*. *africana* and *N*. *attenuata*.

**Table 2:** Concentrations and proportions of the main three sugars in nectar of different *Nicotiana* species.

Species	Concentration of sugars [mM]				Percentages of sugars (calc. from g/l) [%]			Sugar content in nectar (w/v)[%]	Ratio fru/ glu	Suc/ (glu+fru)
	Glucose	Fructose	Sucrose	Total	Glucose	Fructose	Sucrose			
day-flowering										
*N*. *africana*	842 ± 107	820 ± 136	101 ± 41	1763 ± 260	46 ± 2	44 ± 2	10 ± 3	33 ± 5	1.0	0.12
*N*. *attenuata*	531 ± 93	644 ± 110	71 ± 27	1246 ± 212	41 ± 2	49 ± 2	10 ± 3	24 ± 4	1.2	0.11
*N*. *glauca*	49 ± 25	615 ± 155	386 ± 74	1049 ± 180	3 ± 1	44 ± 8	53 ± 8	25 ± 4	12.6	1.10
*N*. *knightiana*	165 ± 65	733 ± 116	572 ± 97	1471 ± 248	8 ± 2	37 ± 3	55 ± 2	36 ± 6	4.4	1.21
*N*. *langsdorffii*	277 ± 104	358 ± 158	405 ± 185	1039 ± 436	20 ± 2	26 ± 3	54 ± 4	25 ± 11	1.3	1.21
*N*. *nudicaulis*	667 ± 201	887 ± 205	430 ± 171	1984 ± 554	28 ± 1	39 ± 5	33 ± 4	43 ± 13	1.3	0.53
*N*. *palmeri*	456 ± 145	646 ± 294	253 ± 86	1355 ± 485	29 ± 3	40 ± 6	31 ± 6	29 ± 10	1.4	0.44
*N*. *paniculata*	409 ± 196	653 ± 204	113 ± 62	1176 ± 446	31 ± 5	53 ± 7	16 ± 4	23 ± 9	1.6	0.20
*N*. *rustica*	237 ± 115	870 ± 162	304 ± 95	1411 ± 323	13 ± 4	53 ± 7	34 ± 5	30 ± 7	3.7	0.52
*N*. *tabacum*	402 ± 29	362 ± 29	247 ± 34	1011 ± 80	33 ± 2	29 ± 1	38 ± 3	22 ± 2	0.9	0.61
*mean*	**398 ± 106**	**654 ± 156**	**285 ± 86**	**1338 ± 318**	**25 ± 2**	**41 ± 4**	**33 ± 4**	**29 ± 7**	**2.9**	**0.61**
night-flowering										
*N*. *acuminata*	485 ± 202	539 ± 226	270 ± 82	1294 ± 564	31 ± 6	34 ± 6	35 ± 11	28 ± 8	1.1	0.50
*N*. *alata*	226 ± 73	656 ± 194	699 ± 108	1581 ± 300	10 ± 3	29 ± 4	61 ± 6	40 ± 7	2.9	1.51
*N*. *benthamiana*	585 ± 170	721 ± 185	181 ± 63	1487 ± 395	35 ± 1	44 ± 3	21 ± 4	30 ± 8	1.2	0.26
*N*. *longiflora*	311 ± 85	466 ± 91	503 ± 112	1281 ± 178	18 ± 5	27 ± 4	55 ± 7	31 ± 5	1.5	1.23
*N*. *nesophila*	44 ± 18	159 ± 45	214 ± 89	417 ± 138	7 ± 2	27 ± 4	66 ± 6	11 ± 4	3.6	2.00
*N*. *otophora*	316 ± 48	270 ± 44	169 ± 47	756 ± 88	35 ± 3	30 ± 5	35 ± 8	16 ± 2	0.9	0.55
*N*. *plumbaginifolia*	372 ± 128	421 ± 127	178 ± 103	971 ± 340	33 ± 3	38 ± 6	28 ± 6	20 ± 8	1.1	0.43
*N*. *stocktonii*	106 ± 29	300 ± 56	178 ± 36	585 ± 110	14 ± 3	40 ± 1	45 ± 4	13 ± 2	2.8	0.83
*N*. *suaveolens*	648 ± 217	677 ± 250	113 ± 53	1437 ± 496	42 ± 3	44 ± 3	14 ± 5	28 ± 9	1.0	0.16
*N*. *sylvestris*	294 ± 77	328 ± 74	455 ± 110	1077 ± 244	20 ± 3	22 ± 2	58 ± 4	27 ± 6	1.1	1.39
*mean*	**339 ± 105**	**454 ± 129**	**296 ± 80**	**1089 ± 285**	**25 ± 3**	**34 ± 4**	**42 ± 6**	**24 ± 6**	**1.7**	**0.89**

In general, the sucrose-to-hexose ratio was significantly higher in night-flowering species (*p* < 0.001, df = 196, *n* = 198). Glucose’s fraction of total sugar content was similar for day- and night-flowering species (Table 2), whereas the percentage of fructose was significantly higher in day-flowering species (*p* < 0.001, df = 188; *n* = 198). At the same time the percentage of sucrose was increased in night-flowering species; this difference was on a significant level as well (*p* = 0.001, df = 195, *n* = 198).

When including all *Nicotiana* species, the percentage of total sugar content that was sucrose positively correlated with flower tube lengths (r_S_ = 0.451, *p* < 0.001). High percentages of sucrose were found in long-tubed species, e.g. *N*. *alata* or *N*. *longiflora*. Furthermore, the highest percentage of sucrose was found in *N*. *nesophila* with a medium-tubed flower but the smallest diameter of the tube in relation to other species (Table 1). Most of the long-tubed flowers belong to the night-flowering species which are pollinated by hawk moths. The correlation between the percentage of sucrose and the flower tube length is much stronger in night-flowering (r = 0.586, *p* < 0.001) than in day-flowering species, where no correlation exists at all (r = 0.090, *p* < 0.001).

**Fig 2:**
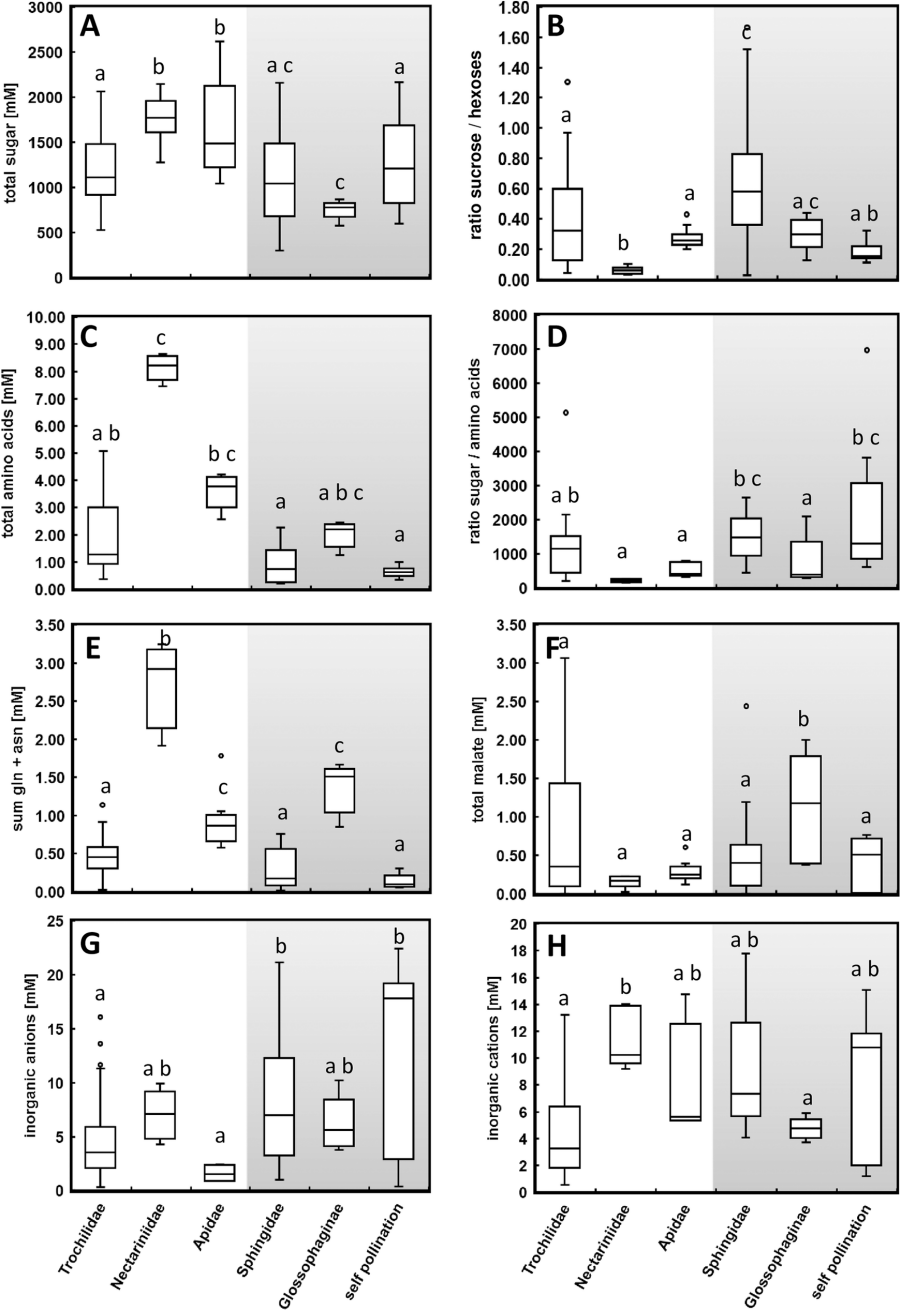
Boxplots of different nectar traits grouped into day- (left, white background) and night-flowering (right, grey background) species. The data are arranged according to their main pollinators Trochilidae, Nectariniidae, Apidae, Sphingidae, Glossophaginae and self-pollinating species. (A) Boxplot diagram illustrating concentration of total sugars [mM]. (B) Sucrose-hexose-ratio is calculated by dividing sucrose concentration [g L^-1^] by the sum of glucose and fructose [g L^-1^]. (C) Boxplots illustrating the concentration of total amino acids [mM]. (D) Ratio sum of sugars-to-sum of amino acids. (E) Concentration of the amides glutamine and asparagine [mM]. (F) Total malate concentrations [mM]. (G) Concentration of the sum of inorganic anions (chloride, nitrate, phosphate, sulphate) [mM]. (H) Concentration of the sum of inorganic cations (potassium, sodium, ammonium, magnesium, calcium) [mM]. Different letters designate significantly different groups determined via ANOVA, post hoc Tukey’s HSD test and Kruskal-Wallis test for non-parametrical data (*p* ≤ 0.05).

Significantly higher sugar concentrations of about 1720 mM (Fig 2A; Table 2) were found in nectar of sunbird- and bee-pollinated species, which corresponds to a share of 35% sugars within nectar (w/v). Nectar of hummingbird-pollinated species generally has a medium concentration with an average of about 1150 mM sugars (26% (w/v)), similar to hawk moth-pollinated and the autogamous species. The nectar of the bat-pollinated species *N*. *otophora* was most diluted with about 750 mM (16% (w/v)).

The highest proportion of hexoses, particularly glucose, was found in the nectar of the sunbird-pollinated species *N*. *africana* (Table 2). Within the hummingbird-pollinated species the proportion of hexoses differed between 45–90%. For hawk moth-pollinated species the proportion of hexoses was, on average, lower than in hummingbird-pollinated species, but also varying between 33–90%. Therefore, the sucrose-to-hexose ratio was lowest in *N*. *africana* (sunbird-pollinated), followed by autogamous species. A medium ratio was found in species pollinated by hummingbirds, bees and bats, and the highest ratio was found in species pollinated by hawk moths (Fig 2B).

To exclude the possibility that the measured differences in sugar composition are a result of microbial activity, the samples were tested for presence of yeast. However, no contaminations with yeast in the nectar samples from the different *Nicotiana* species were found.

The overall sugar content of the leaves was analysed as well in order to disprove that the nectar sugars are not correlated with sugar compositions in leaves. The leaves of all tobacco species also contained glucose, fructose, and sucrose, but the composition of these sugars in the nectar did not correlate with the composition in leaves from the same species (S1 Fig).

### Concentrations of amino acids in nectar

The amino acid concentrations were much lower than the sugar concentrations and also different between the *Nicotiana* species. Table 3 shows percentages of the most abundant amino acids, the essential amino acids, and total concentrations of the 19 proteinogenic amino acids that were detectable (alanine (*ala*), arginine (*arg*), aspartate (*asp*), asparagine (*asn*), glycerine (*gly*), glutamate (*glu*), glutamine (*gln*), histidine (*his*), isoleucine (*ile*), leucine (*leu*), lysine (*lys*), methionine (*met*), phenylalanine (*phe*), proline (*pro*), serine (*ser*), threonine (*thr*), tryptophane (*trp*), tyrosine (*tyr*), valine (*val*)), and the non-proteinogenic amino acid γ-aminobutyric acid (*gaba*).

**Table 3:** Amino acid concentrations, proportions of the most abundant amino acids in nectar and the ratio of sugars to amino acids.

Species	Total amino acids [μM]	Percentages of most abundant amino acids in nectar [%]						Percentage of essential amino acids [%]	Sum sugar [mM]/ sum amino acids [mM]
		Glu	Gln	Asp	Asn	Pro	Ser		
day flowering									
*N*. *africana*	8148 ± 423	1 ± 0	31 ± 4	1 ± 0	2 ± 1	36 ± 5	6 ± 1	17 ± 3	216
*N*. *attenuata*	900 ± 288	9 ± 2	49 ± 4	8 ± 1	7 ± 2	5 ± 1	2 ± 0	14 ± 2	1385
*N*. *glauca*	2724 ± 234	4 ± 2	23 ± 4	3 ± 1	2 ± 0	28 ± 3	14 ± 2	11 ± 1	385
*N*. *knightiana*	1157 ± 141	8 ± 1	23 ± 8	7 ± 1	7 ± 4	21 ± 7	10 ± 1	20 ± 5	1271
*N*. *langsdorffii*	3537 ± 538	4 ± 1	11 ± 4	6 ± 2	2 ± 1	34 ± 5	8 ± 1	30 ± 6	294
*N*. *nudicaulis*	3394 ± 667	2 ± 1	25 ± 5	6 ± 3	10 ± 5	18 ± 4	8 ± 4	24 ± 8	585
*N*. *palmeri*	3929 ± 891	2 ± 1	15 ± 5	4 ± 0	3 ± 1	17 ± 5	14 ± 2	39 ± 7	345
*N*. *paniculata*	1199 ± 244	8 ± 2	27 ± 9	10 ± 3	12 ± 8	12 ± 3	7 ± 3	18 ± 6	980
*N*. *rustica*	3700 ± 448	5 ± 1	17 ± 4	53 ± 9	3 ± 2	8 ± 2	3 ± 1	9 ± 4	381
*N*. *tabacum*	600 ± 261	10 ± 5	12 ± 9	23 ± 19	12 ± 7	7 ± 4	7 ± 3	17 ± 5	1685
***mean***	**2919 ± 408**	**5 ± 2**	**23 ± 6**	**12 ± 4**	**6 ± 3**	**19 ± 4**	**8 ± 2**	**20 ± 4**	**753**
night flowering									
*N*. *acuminata*	1428 ± 157	3 ± 1	32 ± 5	2 ± 2	1 ± 1	10 ± 2	2 ± 0	48 ± 7	906
*N*. *alata*	262 ± 44	4 ± 1	28 ± 9	20 ± 9	6 ± 4	6 ± 1	5 ± 2	24 ± 4	6045
*N*. *benthamiana*	707 ± 150	7 ± 1	10 ± 5	42 ± 10	5 ± 3	3 ± 1	5 ± 2	20 ± 3	2103
*N*. *longiflora*	1912 ± 219	5 ± 2	32 ± 5	6 ± 1	2 ± 0	19 ± 4	0 ± 0	34 ± 5	670
*N*. *nesophila*	297 ± 131	9 ± 3	18 ± 8	12 ± 2	1 ± 0	7 ± 3	4 ± 1	44 ± 5	1403
*N*. *otophora*	2019 ± 433	4 ± 1	42 ± 4	4 ± 1	26 ± 4	10 ± 2	6 ± 1	6 ± 1	374
*N*. *plumbaginifolia*	577 ± 205	11 ± 5	23 ± 3	8 ± 2	3 ± 2	8 ± 3	8 ± 2	26 ± 4	1683
*N*. *stocktonii*	961 ± 283	7 ± 1	36 ± 8	16 ± 7	8 ± 4	6 ± 2	3 ± 1	20 ± 4	609
*N*. *suaveolens*	1002 ± 400	7 ± 3	20 ± 9	16 ± 6	4 ± 2	26 ± 9	4 ± 1	21 ± 6	1434
*N*. *sylvestris*	271 ± 53	7 ± 4	20 ± 4	9 ± 2	9 ± 4	5 ± 1	9 ± 2	22 ± 9	3972
***mean***	**944 ± 208**	**6 ± 2**	**26 ± 6**	**14 ± 4**	**6 ± 2**	**10 ± 3**	**5 ± 1**	**26 ± 4**	**1920**

The nectar of *N*. *alata* (night-flowering) contained the lowest concentration, whereas the concentration in *N*. *africana* (day-flowering) was more than 30-fold higher (Table 3). Overall, the total amino acid concentration was significantly higher in nectar of day-flowering species than in night-flowering species (*p* = 0.011, df = 48; *n* = 98). Therefore, the ratio between sum of sugars and sum of amino acids was about 3-fold higher in night-flowering species, mainly due to the lower amino acid concentrations in the nectar of night-flowering species (Table 3).

The percentage of each amino acid was also different between the species (Table 3). Glutamine, proline, and aspartate were the most abundant amino acids in nectar of *Nicotiana* species. Glutamine was found in all species and contributed a substantial proportion of all amino acids in nectar, except for the day-flowering species *N*. *africana*, *N*. *glauca*, *N*. *langsdorffii*, *N*. *palmeri* and the night-flowering species *N*. *suaveolens*, where proline was the dominant amino acid. The amino acid composition in the nectar did not correlate with the composition in the leaves from the same species (S2 Fig).

The essential amino acids for most pollinators are arginine, histidine, isoleucine, leucine, lysine, methionine, phenylalanine, threonine, tryptophane, and valine [18]. The percentage of essential amino acids was between 6% and 48% (Table 3) and similar in day- and night flowering species. However, the concentration of essential amino acids was significantly higher in nectar of day-flowering species than in nectar of night-flowering species (*p* < .001, df = 48; *n* = 98), which was also shown for the total amino acid concentration. The main amino acids in the group of the essential amino acids were valine (in seven species), phenylalanine and lysine (each in four species), histidine (in two species) and arginine as well as isoleucine (each in one species). The relative share of phenylalanine was highly variable in nectar (between less than 1% of the total amino acid concentration in *N*. *glauca* and up to 33% in *N*. *acuminata*). Phenylalanine was only a minor amino acid in leaves of all *Nicotiana* species (about 1–2%; data not shown). In addition to the amino acids normally found in proteins also some non-proteinogenic amino acids were detected in the nectar of *Nicotiana* species, mainly γ-aminobutyric acid (*gaba*). The relative share of *gaba* was low (1–2%) with two exceptions (12% of the total amino acid concentration in *N*. *sylvestris* and 4% in *N*. *tabacum)*.

The amino acid concentrations in the nectar of the *Nicotiana* species differentiated by pollinators are shown in Fig 2C. Nectar of the sunbird-pollinated species *N*. *africana* contained the highest amino acid concentration (8.2 mM) of all day-flowering species, followed by the bee-pollinated species (3.6 mM). For the night-flowering species the highest concentration was measured in the bat-pollinated species *N*. *otophora* (2.0 mM). Therefore, the ratio of total sugars to total amino acids was lower in these pollination types than in hummingbird- and hawk moth-pollinated species, as well as in autogamous species (Fig 2D). Amides (glutamine and asparagine) have a higher nitrogen-to-carbon ratio compared with several other amino acids. The concentrations of amides were very high in the sunbird-pollinated species (mainly glutamine) as well as in the bat-pollinated species (asparagine and glutamine) and in bee-pollinated species (Fig 2E). For the essential amino acids no significant differences between pollinator groups could be shown with the exception of the sunbird-pollinated species *N*. *africana*, which has the highest concentration of essential amino acids in nectar (data not shown). This also applies for the proline concentration (data not shown).

**Fig 3:**
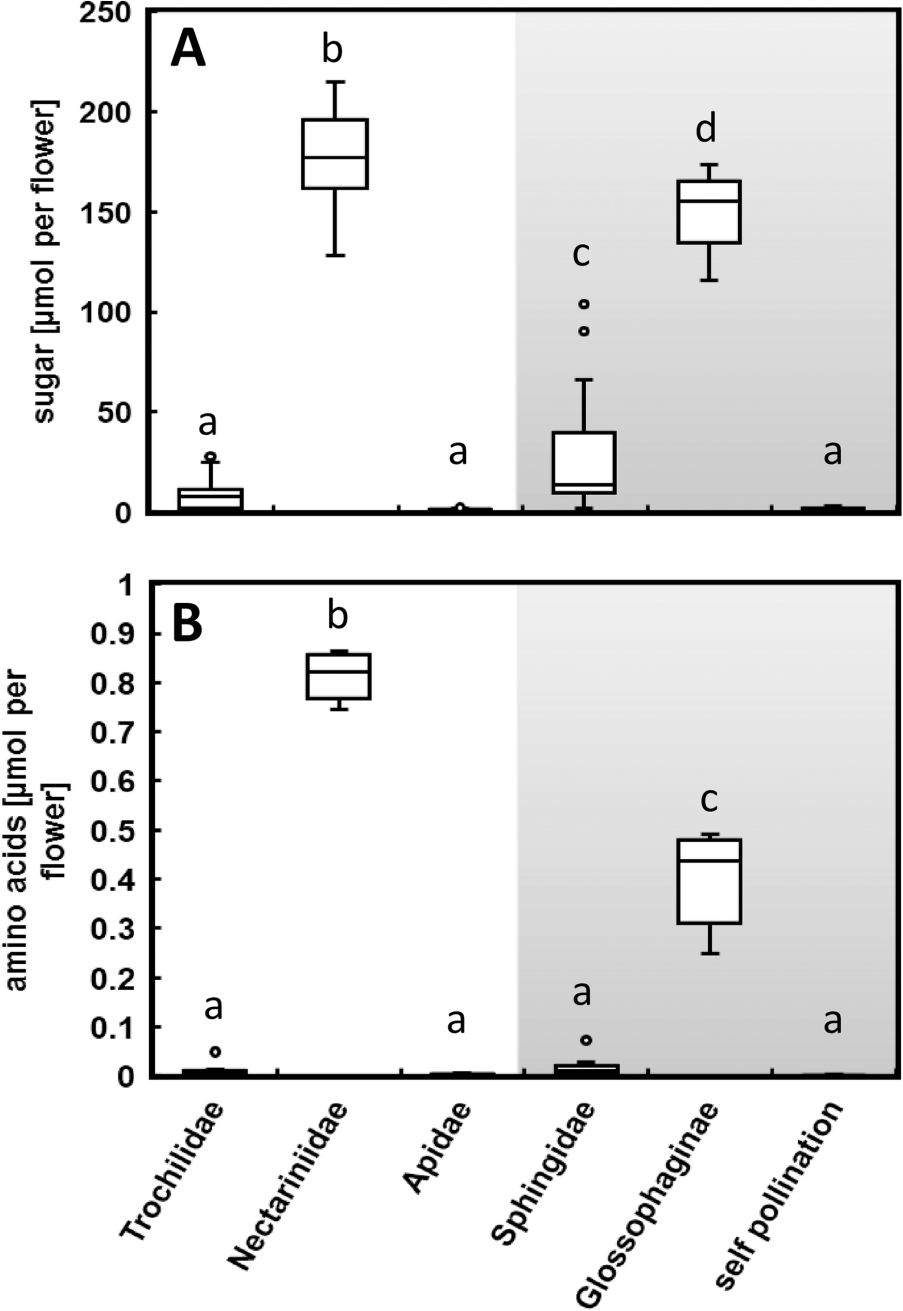
**Boxplots of sugar (A) and amino acid (B) amount in μmol per flower.** Data were calculated by multiplication of the sugar or amino acid concentrations (Table 2 and Table 3) with the approximate nectar volume of each species. Results are grouped by their main pollinators Trochilidae, Nectariniidae, Apidae, Sphingidae, Glossophaginae and self-pollinating species. Different letters designate significantly different groups determined via ANOVA, post hoc Tukey’s HSD test and Kruskal-Wallis test for non-parametrical data (*p* ≤ 0.05).

Flowers of the different tobacco species contained unequal volumes of nectar, from very small volumes in bee- or self-pollinated species (0.5–1 μL), small volumes in hummingbird-pollinated species (1–10 μL), to high volumes in hawk moth-pollinated species (5–50 μL) and abundant nectar volumes in species pollinated by sunbirds or bats (100–200 μL and more). Therefore, the amount of sugars and amino acids per flower between different *Nicotiana* species was also diverse. Sunbird- and bat-pollinated species provided about 150–170 μmol sugars and 0.4–0.8 μmol amino acids per flower (Fig 3). In comparison, the amount of sugar in the nectar per flower of autogamous species was about 100-fold lower (1–1.5 μmol) and the amount of amino acids was even about 1000-fold lower (0.0005 μmol). On the basis of higher nectar volumes of sphingophilous species compared to those of hummingbird-pollinated species, the amounts of sugars and amino acids per flower were higher in hawk moth-pollinated species (Fig 3), although the amino acid concentrations were higher in hummingbird-pollinated species (Fig 2C).

### Concentrations of organic acids in nectar

For the measurement of organic acids and inorganic ions by the method mentioned above, volumes of at least 5 μL per sample are necessary. For some of the less nectar producing species it was not possible to fulfil this requirement, so that *N*. *nudicaulis* and *N*. *stocktonii* are excluded from the analyses. From all other species, at least three and up to five samples could be analysed.

In nectar of *Nicotiana* malate was present in all analysed species, but in different amounts (Table 4). Other organic anions like oxalate and citrate could only be detected in a few samples and in very low concentrations. The lowest concentration of malate was found in hummingbird-pollinated *N*. *palmeri* (0.05 mM) and the highest in *N*. *langsdorffii* (2 mM), which is pollinated by hummingbirds as well. No significant difference between the averaged malate concentrations in day-flowering (0.7 ± 0.2 mM) compared to night-flowering tobacco (0.6 ± 0.2 mM) was obtained.

**Table 4:** Concentrations of malate, inorganic anions and cations as well as the proportions of the single ions in the nectar.

Species	Total malate [mM]	Total anions [mM]	Percentages of anions [%]				Total cations [mM]	Percentages of cations [%]				
			Cl^-^	NO_3_^-^	PO_4_^3-^	SO_4_^2-^		K^+^	Na^+^	NH_4_^+^	Mg^2+^	Ca^2+^
day flowering												
*N*. *africana*	0.1 ± 0.1	3.5 ± 1.4	96 ± 3	2 ± 2	1 ± 1	1 ± 1	11.5 ± 2.0	95 ± 1	3 ± 1	0 ± 0	1 ± 0	0 ± 0
*N*. *attenuata*	1.5 ± 0.6	3.6 ± 1.3	58 ± 13	29 ± 12	5 ± 2	9 ± 3	4.3 ± 1.1	85 ± 4	7 ± 2	0 ± 0	3 ± 0	5 ± 2
*N*. *glauca*	0.2 ± 0	0.5 ± 0.1	71 ± 4	4 ± 5	15 ± 5	10 ± 4	1.0 ± 0.5	91 ± 2	4 ± 1	3 ± 2	4 ± 1	2 ± 0
*N*. *knightiana*	1.8 ± 0.4	3.7 ± 0.7	54 ± 17	4 ± 2	9 ± 3	33 ± 16	1.5 ± 0.6	86 ± 3	10 ± 2	0 ± 0	2 ± 2	2 ± 2
*N*. *langsdorffii*	2.0 ± 0.9	3.4 ± 0.3	82 ± 7	3 ± 1	12 ± 5	3 ± 1	2.5 ± 0.2	78 ± 1	10 ± 2	0 ± 0	7 ± 2	5 ± 2
*N*. *palmeri*	0.1 ± 0.1	6.0 ± 3.2	57 ± 5	5 ± 5	22 ± 4	17 ± 7	6.7 ± 1.5	97 ± 0	2 ± 1	1 ± 1	0 ± 0	0 ± 0
*N*. *paniculata*	0.1 ± 0.1	1.7 ± 1.5	49 ± 2	12 ± 12	21 ± 10	18 ± 3	4.4 ± 2.0	95 ± 2	4 ± 1	4 ± 1	0 ± 0	0 ± 0
*N*. *rustica*	0.2 ± 0	1.7 ± 0.6	59 ± 8	24 ± 5	5 ± 2	12 ± 6	7.8 ± 4.0	94 ± 1	4 ± 1	4 ± 2	1 ± 0	1 ± 0
*N*. *tabacum*	0.4 ± 0.1	12.7 ± 1.9	96 ± 1	1 ± 1	1 ± 1	2 ± 1	9.3 ± 2.9	93 ± 4	5 ± 1	1 ± 0	1 ± 0	1 ± 1
***mean***	**0.7 ± 0.2**	**4.9 ± 1**	**69 ± 7**	**9 ± 5**	**10 ± 4**	**12 ± 5**	**5.6 ± 1.7**	**91 ± 2**	**5 ± 1**	**2 ± 1**	**2 ± 1**	**2 ± 1**
night flowering												
*N*. *acuminata*	0.4 ± 0.2	1.6 ± 0.4	46 ± 6	35 ± 5	7 ± 2	11 ± 3	6.1 ± 1.4	92 ± 1	6 ± 1	1 ± 0	1 ± 0	1 ± 0
*N*. *alata*	0.4 ± 0.1	7.9 ± 1.1	91 ± 5	1 ± 0	1 ± 1	4 ± 2	4.7 ± 0.8	95 ± 1	3 ± 1	1 ± 0	1 ± 0	1 ± 0
*N*. *benthamiana*	0.5 ± 0.2	19.2 ± 1.8	97 ± 1	1 ± 1	1 ± 1	1 ± 1	12.0 ± 1.6	91 ± 1	4 ± 1	0 ± 0	1 ± 0	4 ± 1
*N*. *longiflora*	0.1 ± 0	14.3 ± 2.5	98 ± 2	1 ± 1	0 ± 0	1 ± 1	12.4 ± 4.7	95 ± 2	2 ± 1	1 ± 0	2 ± 2	0 ± 0
*N*. *nesophila*	1.1 ± 0.1	17.1 ± 2.6	97 ± 0	0 ± 0	1 ± 0	2 ± 1	10.3 ± 4.4	90 ± 2	9 ± 2	3 ± 1	1 ± 0	0 ± 0
*N*. *otophora*	1.6 ± 0.6	6.1 ± 2.3	59 ± 5	39 ± 5	0 ± 0	1 ± 1	4.7 ± 0.7	97 ± 1	1 ± 0	0 ± 0	2 ± 0	0 ± 0
*N*. *plumbaginifolia*	0.2 ± 0.3	3.5 ± 4.2	93 ± 5	1 ± 1	3 ± 1	4 ± 4	1.9 ± 0.6	89 ± 2	8 ± 2	0 ± 0	1 ± 0	1 ± 1
*N*. *suaveolens*	0.3 ± 0.2	6.0 ± 1.2	84 ± 9	1 ± 1	10 ± 6	5 ± 3	14.0 ± 2.3	97 ± 0	2 ± 0	1 ± 0	1 ± 0	1 ± 0
*N*. *sylvestris*	0.4 ± 0.2	10.4 ± 1.8	94 ± 1	2 ± 1	1 ± 0	3 ± 1	9.1 ± 2.1	99 ± 0	0 ± 0	0 ± 0	1 ± 0	0 ± 0
***mean***	**0.6 ± 0.2**	**10.2 ± 1.9**	**84 ± 4**	**9 ± 2**	**3 ± 1**	**4 ± 2**	**8.4 ± 2.2**	**94 ± 1**	**3 ± 1**	**1 ± 0**	**1 ± 0**	**1 ± 0**

Malate concentrations in the nectar of the *Nicotiana* species differentiated by pollinators are shown in Fig 2F. With the exception of the bat-pollinated species *N*. *otophora* no significant differences in malate concentrations were found between the pollinator groups.

Almost no correlation was found between the malate content in leaves and nectar of all tobacco species (*R*^*2*^ = 0.149, *p* = 0.004) (S3 Fig).

### Concentrations of inorganic ions in nectar

There were large differences in the total concentration of inorganic anions, that ranged from a minimum of 0.5 ± 0.2 mM in *N*. *glauca* to a maximum of 19.2 ± 1.8 mM in *N*. *benthamiana* (Table 4). Chloride was the most abundant anion in all analysed species and represented a minimum share of 45 ± 7% of the total inorganic anions in *N*. *acuminata* and a maximum share of 98 ± 2% in *N*. *longiflora*. Nitrate, phosphate, and sulphate were present in all species but on a lower level. Overall, the total inorganic anion concentration was about 2-fold higher in nectar of night-flowering species than in day-flowering species (*p* = 0.05, df = 9; *n* = 99). The total concentrations of inorganic anions differentiated by pollinators are shown in Fig 2G. Due to the higher concentration of anions in nocturnal species in general, the nocturnal pollinator groups can also be distinguished from the pollinator groups active during daytime.

*N*. *otophora*, the only bat-pollinated plant, produces the most nitrate containing nectar. The percentage of nitrate made up 38% of the total anion content, whereas for the other species the percentage of nitrate ranged from less than 1% in sphingophilous *N*. *nesophila* to 32% in ornitophilous *N*. *attenuata*. Several hummingbird-pollinated species contained sulphate- and phosphate-rich nectar. Sulphate accounts for 33% of the measured anions in *N*. *knightiana* and phosphate accounts for one quarter of the anions in *N*. *palmeri*. As mentioned before the inorganic anion concentration was measured for the tobacco leaves in parallel (S4 Fig). Hardly any correlation was found between the total concentration of anions or the single anions in nectar and leaf samples (*R*^*2*^ = 0.006, *p* < 0.001).

The total concentration of inorganic cations was similar to the concentration of inorganic anions (Table 4). The concentration ranged from a minimum of 1 mM in *N*. *glauca* to a maximum of 14 mM in *N*. *suaveolens*. Based on total inorganic cation concentration, the relative amounts of potassium varied between 78 ± 1% in *N*. *langsdorffii* and 99 ± 2% in *N*. *sylvestris*. The next most frequent cation was sodium. Its relative proportion was highly variable in nectar (between less than 1% of the total cation concentration in *N*. *sylvestris* and up to 10% in *N*. *knightiana* and *N*. *langsdorffii*). Ammonium, magnesium, and calcium were present in all species but on a lower level. It was not possible to statistically differentiate between day- and night-flowering tobacco plants on the basis of inorganic cation concentrations, even though the mean concentration of all cations was slightly higher in night-flowering compared to day-flowering species. The inorganic cation concentrations differentiated by pollinators are shown in Fig 2H. Nectar of the sunbird-pollinated species *N*. *africana* contained the highest cation concentration whereas the lowest concentration was measured in hummingbird-pollinated species and in the bat-pollinated species *N*. *otophora*.

Overall, the concentration of both inorganic anions and cations was about 10-fold higher than the concentration of organic acids or amino acids, whereas sugars were by far the most dominant compounds in the nectar of all *Nicotiana* species (about 100-fold higher concentrated than inorganic ions).

### Pollinators’ preferences

**Fig 4:**
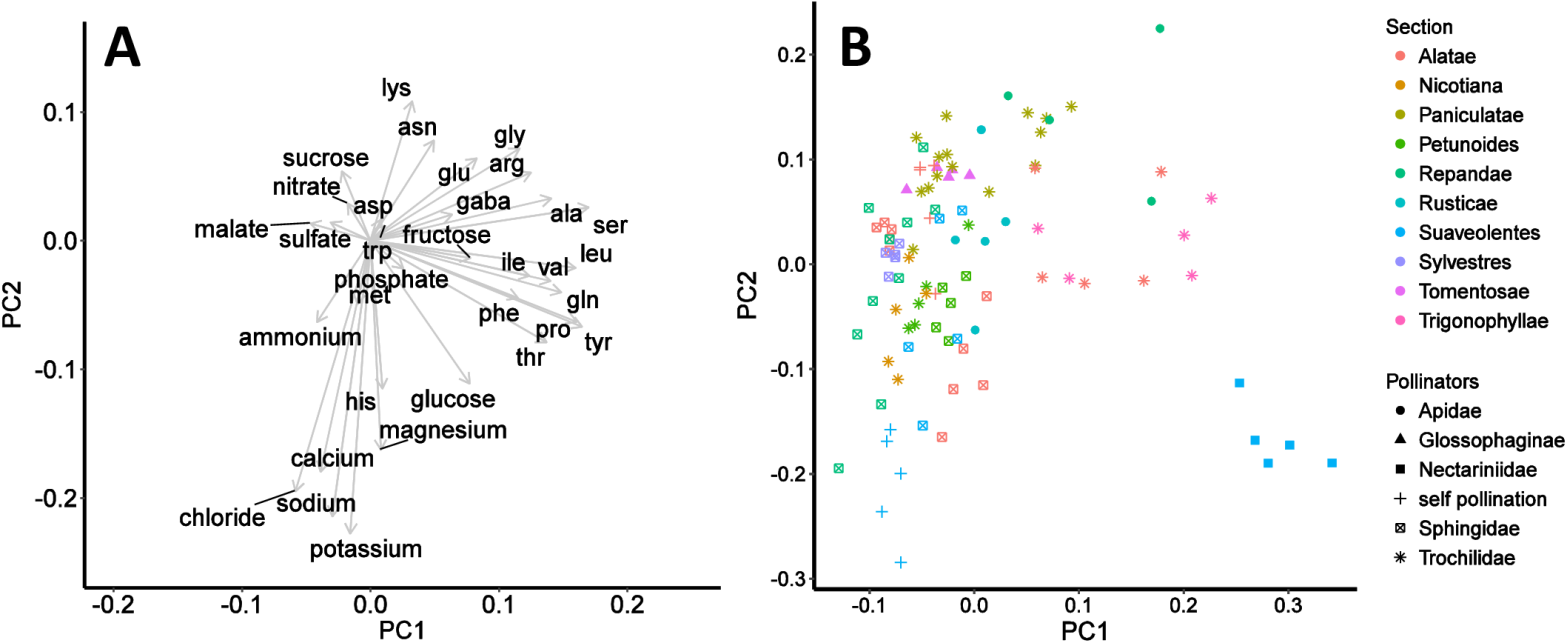
Loadings and scatterplot of PCA scores in rotated space. (A) Loadings of the original variables are shown as vectors in PCA space (B) The first principal component (PC 1) accounts for 24.4% and the second principal component (PC 2) accounts for 10.3% of the variation in the dataset. Eigenvalues are 2.837 for PC 1 and 1.846 for PC 2. Data (*n* = 99) are grouped both by main pollinators (colours) and sections (markings).

To reduce the complexity of the data, a PCA was performed with independently measured nectar features as described in the methods section. Fig 4A shows the loadings of all measured nectar constituents on the extracted principal components. Most of the cations load strongly negative on the first component (K^+^, Na^+^, Mg^2+^, Ca^2+^). The majority of amino acids loads positively on the second component. The first principal component explains 24.4% and both principal components together explain 34.7% of the total variance. Fig 4B shows a scatterplot of the PCA scores with focus on the distribution by pollinator groups and sections, which are representing the phylogenetic constraints of the species. Some groups (especially pollination types) are more distinguishable than others, for example sunbird-pollinated plants (filled box), which cluster in the lower right quadrant and bee-pollinated plants (filled circles), which accumulate in the upper right quadrant. To complement the graphical evaluation, a PERMANOVA was performed on the nectar data with pollinators and sections as categorical variables (Table 5). If all chemical classes (sugars, amino acids, inorganic anions, inorganic cations, and malate) were taken into account, there is a highly significant (*p* < 0.001) difference between the individual pollinator groups and between the individual sections. In this model, 27% of the data variation can be explained by pollinators, 24% by sections, 21% by a combination of both variables, and 27% by other factors. If only sugars and amino acids are taken into account, the distribution was more different: grouping by pollinators is responsible for 66% of the variation whereas section grouping makes up 19% of the variation (*p* < 0.001 each). When the PERMANOVA was performed solely on the measurements of inorganic anions, inorganic cations, and malate, 26% of the data variation could be explained by pollinators and 24% by sections (*p* < 0.001 each; 21% pollinators × sections, 28% residuals).

**Table 5:** Results of the PERMANOVA: Degrees of freedom (df), pseudo-F (F), R^2^, and *p*-value (P).

PERMANOVA	Df	F	R^2^	*p*
a) All components				
Pollinator	5	16.12	0.27	0.001***
Section	8	8.94	0.24	0.001***
Pollinator x Section	2	31.31	0.21	0.001***
Residuals	83		0.28	
Total	98		1.00	
b) Sugars and amino acids				
Pollinator	5	85.55	0.66	0.001***
Section	8	15.56	0.19	0.001***
Pollinator x Section	2	8.70	0.03	0.001***
Residuals	83		0.13	
Total	98		1.00	
c) Anions, cations, and malate				
Pollinators	5	15.05	0.26	0.001***
Section	8	8.83	0.24	0.001***
Pollinator x Section	2	31.66	0.22	0.001***
Residuals	83		0.28	
Total	98		1.00	

## Discussion

We are presenting here the first comprehensive study about the content of sugars, amino acids, organic acids, and inorganic ions in nectars of a large set of species from one genus comprising a wide variety of pollinators. A simplified phylogenetic tree demonstrates that the selection of pollinators is independent from the sectional grouping of the *Nicotiana* plants tested here (S5 Fig). Key factors exhibiting a significant influence on visitation of pollinators to plants include nectar production and the composition of nectar with respect to sugars, amino acids, organic acids, inorganic ions, and other metabolites. Correlations between nectar sugar compositions and pollinator preferences have been demonstrated in several previous studies [8,45,56,57]. However fewer studies investigated a relationship between nectar amino acids and pollinators [13,14] and studies focused on organic acids or inorganic ions and pollinator preferences are scarce.

### Sucrose rich nectar correlates with corolla tube length in night-flowering species

Nectar sugar composition differed among all 20 analysed *Nicotiana*. Reasons for differences in nectar composition have not been fully determined so far. Some studies proved that long-tubed flowers with concealed nectaries tend to be associated with sucrose-dominated nectar [1,9,27,58]. This correlation is strongly supported by our findings in night-flowering species (r = 0.586), in contrast to day-flowering species, for which no correlation was found (r = 0.090).

The majority of the night-flowering species are long-tubed tobacco species and therefore they are mainly pollinated by moths. They typically have a very long proboscis supporting the hypothesis that flower shapes have co-evolved with the morphology of the mouth parts of their pollinators [23,24]. Particularly within the group of sphingophilous tobacco plants there exists a very high correlation between the corolla tube lengths and the proportions of sucrose (r = 0.857). *N*. *nesophila* was however excluded due to the fact of exhibiting extreme slender bottle-neck flowers compared to other hawk moth-pollinated flowers (Fig 1). If *N*. *nesophila* was included, the correlation was much lower (r = 0.397). Therefore, sucrose-rich nectar in long-tubed flowers could be an adaptation to the preference of long-tongued pollinators, along with the fact that sucrose-rich nectar is better protected against evaporation in these flowers [9]. Sucrose-dominated sugar solutions tend to evaporate faster than hexose-dominated sugar solutions due to their lower osmotic potential. In addition to the longer tube, higher humidity and lower temperatures at nights may partially prevent evaporation. One benefit of an increased sucrose proportion is the lower viscosity of the nectar. If the viscosity is too high, it will prevent pollinators from extracting nectar and avoid these flowers eventually [29]. Possibly nectar viscosity is aligned with the length of the corolla tube so that effective nectar drinking by hawk moths is possible. Moths are active suction feeders and therefore effective drinking is ensured by nectar with lower sugar concentrations [11].

### Nectar of several Nicotiana species contains more fructose than glucose

Hexose-rich nectar occurs in several *Nicotiana* species (*N*. *africana*, *N*. *attenuata*, *N*. *benthamiana*, *N*. *paniculata*, *N*. *suaveolens*). Hexoses are typically not part of the phloem sap of plants [39] and therefore the proportion of hexoses in nectar depends on the presence and activity of cleaving enzymes in nectaries, including invertases [59].

The proportions of glucose and fructose in nectar of the different *Nicotiana* species were either similar or fructose dominated (Table 2). In eight out of twenty species the fructose-to-glucose ratio was higher than 1.5. The diurnal species *N*. *glauca* exhibited an extremely high ratio of 12.6 followed by *N*. *rustica* with a ratio of 3.7 (Table 2). Also, higher percentages of fructose in comparison to glucose were found in nectars of other plant families, e.g. in some Acanthaceae [60] and Scrophulariaceae [61]. In *Conophytum* species (Aizoaceae) nectar of diurnal species had significantly higher fructose-to-glucose ratios than nectar from nocturnal species [29]. Increased sweetness of fructose rich nectars may be more rewarding for pollinators and therefore provide a natural advantage for plants with higher fructose-to-glucose ratios. Honey bees preferred fructose rather than glucose in artificial nectar as demonstrated by Waller [62], which corresponds to our observation that bee-pollinated *Nicotiana* species (*N*. *nudicaulis*, *N*. *rustica*) contained more fructose as glucose in their nectar (Table 2).

Another explanation for the non-stoichiometric hexose ratio in some tobacco plants could be the result of a yeast contamination caused by nectar probing flower visitors [63]. However, we found no contamination with yeast in the nectar samples of the tested *Nicotiana* species, which confirms the fructose-to-glucose ratio to be genuine.

### Nectar amino acid composition is highly specific for pollinator groups

The presence of amino acids in nectar has been known for several decades, but their role in nectar is still a matter of debate [14,64]. At least two possible explanations for the species-specific differences in nectar amino acid concentration exist: (1) amino acids are leaching from the nectaries and the nectar composition reflects the amino acid composition of the phloem and nectaries or (2) the amino acid composition in nectar is correlated to the preferences of different pollinators. In the latter case amino acids in nectars could present either a potential source of amino acids in the nutrition of the pollinators or the presence of amino acids in nectar potentially contributes to its taste [18,65].

Maximum concentrations in tobacco nectar were about 1500 mM sugars and 8 mM free amino acids (Tables 2 and 3), whereas in the phloem sap about 500 sugar (exclusively sucrose) and 80 mM [66] are transported into the nectaries. Therefore, the carbon-to-nitrogen ratio is clearly higher in the nectar compared to the supplying phloem sap. A discrepancy of amino acids concentration and their composition between phloem sap and nectar was also shown in other plants [39]. This may indicate an active regulation mechanism in the nectaries in order to accumulate sugars and retain amino acids as well as the selective secretion of specific amino acids into the nectar.

All ten essential amino acids for nectarivorous pollinators were present in the nectar of *Nicotiana* species, but glutamine was the predominant amino acid, followed by proline, aspartate, and serine (Table 3). Proline and glutamine were also the dominant amino acids in the nectar of the section Alatae [31] and in most of 30 species from different plant families [13] reported.

Studies with insect preference tests could show a preference of honey bees (*Apis mellifera*) for proline-enriched artificial nectar as well as a negative response to serine [67]. The percentage of proline in the bee-pollinated species *N*. *rustica* and *N*. *nudicaulis* was in the middle range (8% and 17%, respectably, Table 3) whereas the highest percentage of proline was found in the sunbird-pollinated species *N*. *africana* (36%). In floral nectar of 73 Mediterranean plant species, the proportion of phenylalanine was highly variable [14]. This corresponds to the findings in the genus *Nicotiana*. Nectar of *N*. *acuminata*, *N*. *palmeri* and *N*. *longiflora* contained a high proportion of phenylalanine (up to 33% of the total amino acid concentration), whereas the proportion was very low for other species. We found no correlation between phenylalanine concentration in nectar and flowering time, pollination time, section or phenylalanine content in leaves. Further studies are therefore needed to investigate the role of phenylalanine in nectar of certain species.

The amount of amino acids per flower in sunbird- and bat-pollinated species was about 1000-fold higher than in autogamous species and about 220-fold higher than in bee-pollinated species (Fig 3). The concentration of amides (amino acids with a higher nitrogen-to-carbon ratio in comparison to several other amino acids) was particularly high for sunbird- and bat-pollinated species (Fig 2E). As a conclusion, sunbird- and bat-pollinated species either have a large loss of organic nitrogen, or invest large amounts of organic nitrogen for specific pollination by sunbirds or bats. In addition, pollinators could dislodge pollen into the nectar while collecting it, which would again increase the amino acid amount [2]. Generally, Nectariniidae are bigger than hummingbirds [68,69] and therefore might need a higher intake of amino acids, which could provide an explanation for the lower amino acid concentration in hummingbird-pollinated species. This dichotomy in amino acid concentrations in ornithophilous species with lower concentrations in hummingbird-pollinated species and higher concentrations in species pollinated by passerine birds has already been shown by Baker and Baker [2]. In comparison, sunbird-pollinated species have nectar which is the richest in amino acids when compared to all other day-flowering species, whereas bat-pollinated species have the highest amino acid concentrations of all night-flowering species. This leads to the simplified conclusion, that the bigger the pollinators are in size, the more a plant has to invest in their amino acid nutrition.

Hawk moths lack an alternative protein source [2], which is why the low amino acid concentration in nectar of flowers usually pollinated by these moths is surprising. A conceivable explanation is that the collection of relatively large amounts of nectar per night may compensate the low amino acid concentration in the nectar. This would correspond to the higher amount of amino acids per flower in hawk moth-pollinated species in contrast to hummingbird- or bee-pollinated species (Fig 3) as a consequence of larger nectar volumes in hawk moth-pollinated species. All amino acids that are essential for the growth of adult honey bees [70] were detected in nectar of melittophilous *Nicotiana* species except methionine which was under the detection limit. However, bees appear to not solely rely on nectar for their intake of nitrogen and essential amino acids. Pollen seems to represent an additional source. As a consequence bees are not as dependent on free amino acids from nectar as expected [70].

### The function of organic acids in nectar

Organic acids in nectars have not been studied in detail, despite the fact that they may play an important role in nectar quality and pollinator attraction, e.g. by adding flavours and aromas to the nectar [16]. The main organic acid in nectars of all analysed *Nicotiana* species was malic acid and malate respectively, followed by citrate. This result corresponds to the composition of organic acids in the nectar of *Aquilegia *[16]. The concentration of malate in nectars of *Nicotiana* species was between 0.1 and 2.0 mM, similar to levels found in apoplastic fluids [41], but considerably lower than concentrations in leaves (approx. 10 μmol g^-1^ FW^-1^ and 200 μmol g^-1^ FW^-1^ which corresponds to about 12 to 235 mM considering the aqueous space of the leaves of about 85%). An explanation for this concentration gradient may be the fact that malate has no apparent benefit for the pollinators like sugars or amino acids and therefore the plants may limit the output of this organic acid. This could also explain why the concentration of malate in the nectar did not change significantly along with the type of pollinator (Fig 2F), except for the bat-pollinated species *N*. *otophora*. However, it cannot be excluded that organic acids may play a role in pollinator attraction, e.g. by adding flavours to the nectar [16].

### Inorganic ions are higher concentrated in the nectar of night-flowering species and organic metabolites are higher concentrated in the nectar of day-flowering species

Nectar of night-flowering species is generally more dominated by inorganic ions than nectar of day-flowering species (inorganic anions are 2-fold higher and inorganic cations are 1.5-fold higher, Table 4). On the other hand, nectar of day-flowering species contained about 20% more sugar (Table 2) and the total amino acid concentration was about 3-fold higher in nectar of day-flowering species (Table 3). The reason of the lower sugar and amino acid concentration in nectar of night-flowering species could be the lower assimilation rate of carbon and nitrogen in the whole plant and the lower phloem translocation rate of assimilates during the night phase [71]. The processes leading to higher concentrations of inorganic ions in nectar of night-flowering species are still poorly explored and further analyses are required.

The detected spectrum of inorganic ions is comparable to levels found in apoplastic fluid, while inorganic ion concentration is usually higher in the symplast [41]. In each case chloride and potassium were the main anion and cation, respectively. Nitrate is far less concentrated in the nectar than in the apoplastic fluid. This might be due to a regulatory mechanism preventing ions from being secreted into the sugary solution. Much higher potassium than sodium concentrations in nectar are according to concentrations of these cations in the phloem sap [72].

Ion concentration in nectar influences the electrolyte balance of nectar-feeding birds [19]. The Broad-tailed Hummingbird (*Selasphorus platvcercus*) for example needs to replace 14% of its body electrolytes each day [73]. So far, no data on electrolyte balance are available for other pollinator groups. Hiebert and Calder found average chloride concentrations of 9.9 mM in 19 hummingbird-pollinated species, which is close to our findings in *Nicotiana* (5.8 mM) [74].

### Nectar volumes and concentrations are adapted to the requirements of the pollinators

The species of *Nicotiana* showed variation in their phenology over the course of the day, thus regulating the availability of nectar to pollinators. Furthermore, nectar volume and composition of nectar are likely to be adapted to the nutritional and energetic requirements of the pollinators [8]. We found that several nectar characteristics in tobacco corresponded to their pollination type when pollinators are specialized to visit specific plants. Several hummingbird-pollinated *Nicotiana* species secreted sucrose-rich nectar, whereas the nectar of the sunbird-pollinated *N*. *africana* was hexose-rich (Fig 2B, Table 2). This dichotomy in bird-pollinated species was shown by Baker and Baker [8] and it may reflect differences in bird physiologies, e.g. different levels of sucrase activity in several nectarivorous perching birds [75,76], and a pattern of nectar secretion, e.g. invertase activity of the nectaries [77]. These data are in agreement with findings of Martinez del Rio [75], who demonstrated an experimental behavioural preference for sucrose over hexoses for some hummingbird species. Napier *et al*. have shown that the preference is depending on the sugar concentration and nectar feeding birds only preferred hexose solutions with low sugar concentrations [76]. The question why nectars of hummingbird-pollinated species are often sucrose dominated and those of sunbird-pollinated species are often hexose dominated is still unresolved, and perhaps a combination of both bird and plant physiologies is involved [77]. Furthermore, in other plant families the sugar composition in nectar was more linked to the phylogeny of the species than to pollinator preferences, e.g. in 35 Asteraceae species which were most visited by numerous insects [27].

Nectar volume is expected to correlate with the body size of the pollinators [18]. The abundant nectar volumes of sunbird- (*N*. *africana*) and bat-pollinated species (*N*. *otophora*) constitute a significant investment of the plants. The amount of sugars per flower was about 150-fold higher in these species than in bee-pollinated species or autogamous species. This difference is even more pronounced for amino acids per flower (up to 1000-fold higher). Conversely, autogamy may facilitate the evolution of reduced nectar volumes as well as nectar concentrations, particularly that of amino acids, due to the decreased need for pollinator attraction. Sugar and amino acid amounts were estimated for individual flowers, rather than for the entire plant. It is possible that differences in sugar and amino acid amounts per flower could be balanced across species by differences in the number of flowers produced along the flowering season [31].

The PCA resulting in a dimensionality reduction allowed us to visualize the distribution of the data. By means of different markings a pattern of the data distribution became partially visible, which led to the conclusion, that phylogenetic constrains and particularly pollination types are suitable to make predictions on nectars’ chemistry. These observations correlate to findings of Petanidou *et al*. [78]. They demonstrated that phylogenetic affinity plays a secondary role, if a PCA is run on the basis of nectar characteristics (nectar volume, sugar and amino acids content) similar to those used by our group. Additionally it was shown for the section Alatae that nectar volume and concentration tend to be more similar among species with the same predominant pollinator compared to species with different predominant pollinators [31].

The PERMANOVA confirmed a significant difference between both the pollination groups and the sections (Table 5). If all measured nectar components are included, the influence of pollinators and sections on the data variance is similar. If only sugars and amino acids are taken into account, the influence of the pollinators becomes dominant. However, the importance of pollinators over sections vanishes if only organic acids and inorganic ions are considered.

It may be concluded that the composition of sugars and amino acids in nectar of *Nicotiana* species is highly influenced by the main pollinator of a plant and that there is a fewer but still significant influence on inorganic ions and malate. Nevertheless, a considerable part of the variance cannot be explained by either of the grouping options, which raises the question if there are further models to predict the nectar composition.

Within the genus *Nicotiana* highly specialized plant species have evolved, the flower morphology and several nectar features of which are aligned to the needs of its pollinators like the sunbird-pollinated species *N*. *africana* and the bat-pollinated species *N*. *otophora*. This evolutionary process did not apply to all examined *Nicotiana* species, resulting in generalists which are accessible to a more diverse group of pollinators. Summarizing all data, it appears that sugar and amino acid concentrations in nectar of *Nicotiana* are primarily influenced by the pollinators of the plant. Other factors such as phylogenetic relationships are less important determinants.

## Supporting information

S1 Fig Total sugar contents in leaves and in nectar of the same *Nicotiana* species. (PDF)

S2 Fig Total amino acid contents in leaves and nectar of the same *Nicotiana* species. (PDF)

S3 Fig Total concentration of malate in leaves and nectar of the same *Nicotiana* species. (PDF)

S4 Fig Total inorganic anion contents in leaves and nectar of the same *Nicotiana* species. (PDF)

S5 Fig Simplified phylogenetic tree of all examined *Nicotiana* species and their main pollinators. (PDF)
