# Supplementary material for: Nectar sugars and amino acids in day- and night-flowering Nicotiana species are more strongly shaped by pollinators’ preferences than organic acids and inorganic ions
Source: PLoS One. 2017 May 3;12(5):e0176865. doi: 10.1371/journal.pone.0176865 (PMC5415175; doi:10.1371/journal.pone.0176865)
Supplement: S1 Fig — (PDF) [file pone.0176865.s001.pdf]

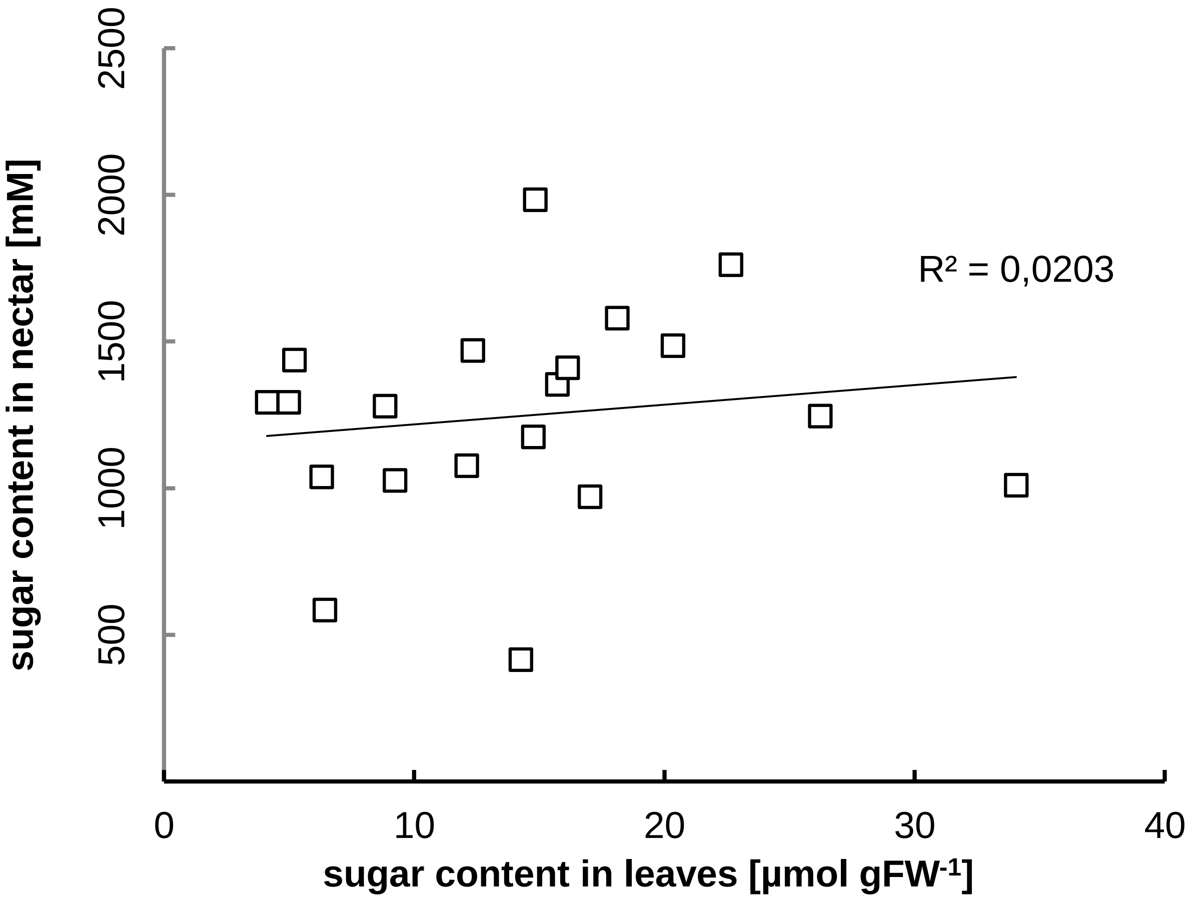

S1 Fig. Total sugar contents in leaves [mmol gFW^{-1}] and in nectar [mM] of the same *Nicotiana* species. Neither does a noteworthy correlation exist between the total concentrations in nectar and leaves (R^2 = 0.02, p = 0.108) nor between the percentages of the main sugars in leaves and in nectar, e.g. glucose (R^2 = 0.005, p = 0.020), fructose (R^2 = 0.001, p = 0.130), and sucrose (R^2 = 0.073, p < 0.001).
