# Supplementary material for: Nectar sugars and amino acids in day- and night-flowering Nicotiana species are more strongly shaped by pollinators’ preferences than organic acids and inorganic ions
Source: PLoS One. 2017 May 3;12(5):e0176865. doi: 10.1371/journal.pone.0176865 (PMC5415175; doi:10.1371/journal.pone.0176865)
Supplement: S2 Fig — (PDF) [file pone.0176865.s002.pdf]

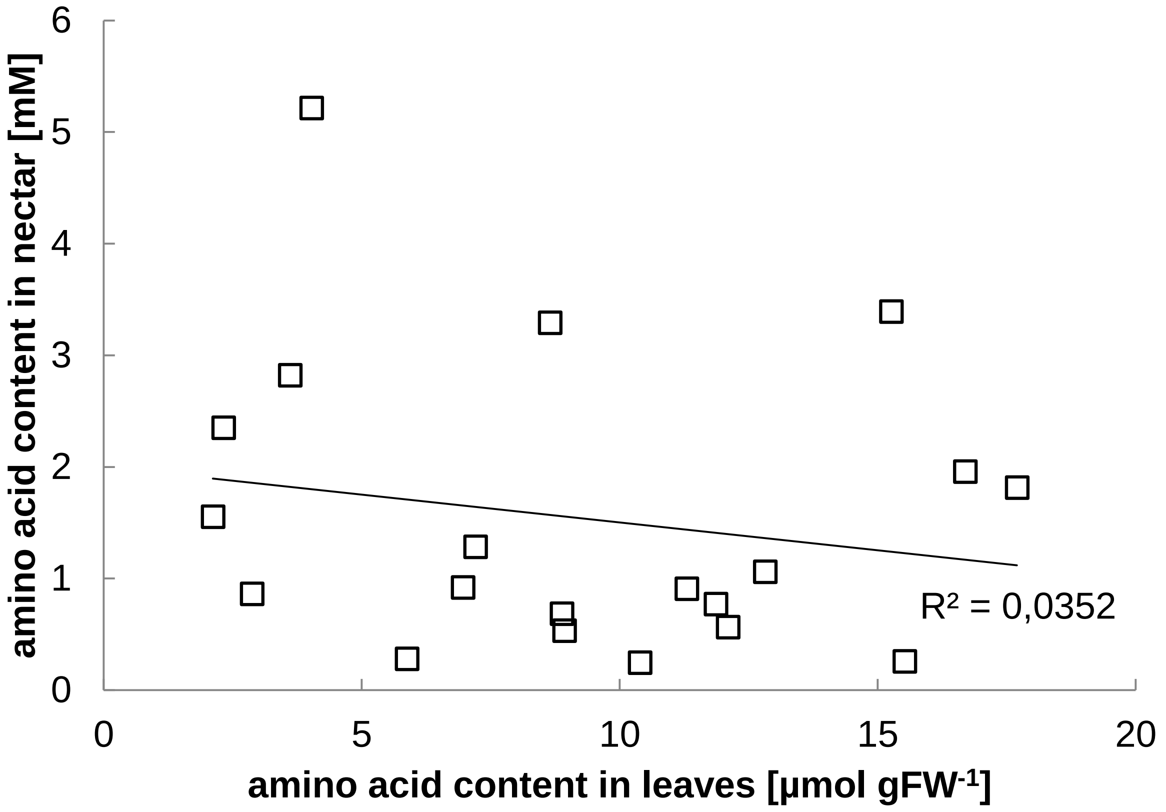

S2 Fig. Total amino acid contents in leaves [µmol gFW^{-1}] and nectar [mM] of the same *Nicotiana* species. Neither does a noteworthy correlation exist between the total concentrations in nectar and leaves (R^2 = 0.035, p < 0.001) nor between the percentages of single amino acids in leaves and nectar, e.g. glutamate (R^2 = 0.011, p < 0.001), glutamine (R^2 = 0.053, p < 0.001), aspartate (R^2 = 0.015, p < 0.001) and asparagine (R^2 = 0.075, p = 0.211).
