# Supplementary material for: Nectar sugars and amino acids in day- and night-flowering Nicotiana species are more strongly shaped by pollinators’ preferences than organic acids and inorganic ions
Source: PLoS One. 2017 May 3;12(5):e0176865. doi: 10.1371/journal.pone.0176865 (PMC5415175; doi:10.1371/journal.pone.0176865)
Supplement: S3 Fig — (PDF) [file pone.0176865.s003.pdf]

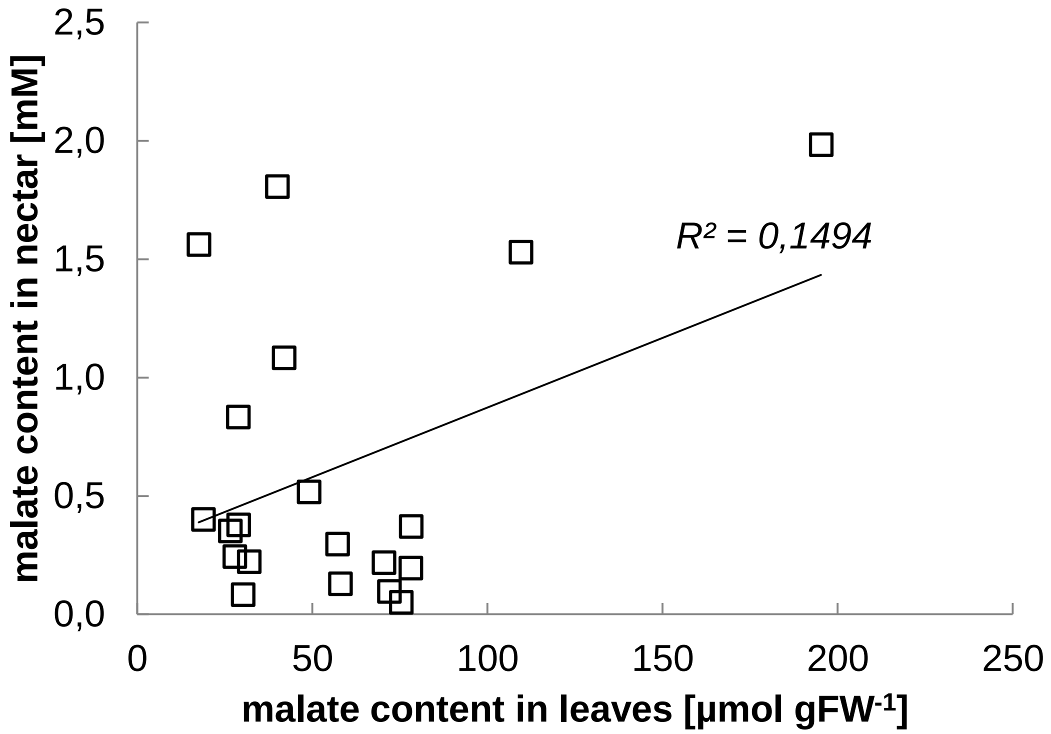

S3 Fig. Total concentration of malate in leaves [µmol gFW^{-1}] and nectar [µmol L^{-1}] of the same *Nicotiana* species. There is almost no correlation between the total concentrations in nectar and leaves (R^2 = 0.149, p = 0.001).
