# Supplementary material for: Nectar sugars and amino acids in day- and night-flowering Nicotiana species are more strongly shaped by pollinators’ preferences than organic acids and inorganic ions
Source: PLoS One. 2017 May 3;12(5):e0176865. doi: 10.1371/journal.pone.0176865 (PMC5415175; doi:10.1371/journal.pone.0176865)
Supplement: S4 Fig — (PDF) [file pone.0176865.s004.pdf]

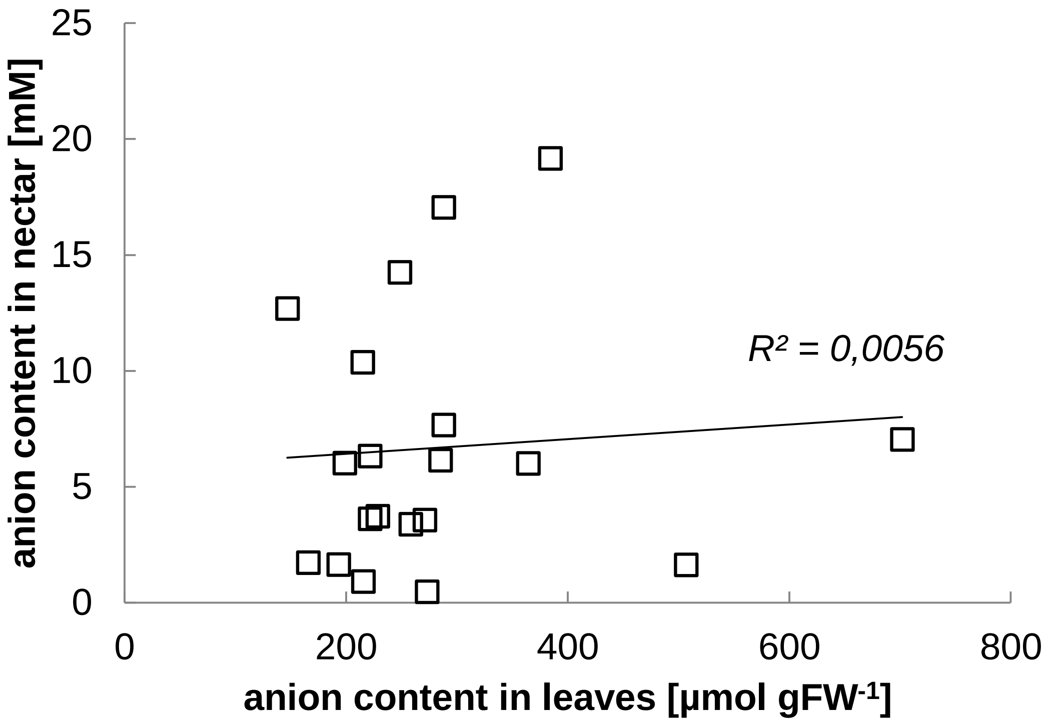

S4 Fig. Total concentration of anions in leaves [mmol gFW^{-1}] and nectar [mM] of the same *Nicotiana* species. Neither does a noteworthy correlation exist between the total concentrations in nectar and leaves (R^2 = 0.006, p < 0.001) nor between the percentages of single anions in leaves and nectar, e.g. chloride (R^2 = 0.221, p = 0.010), nitrate (R^2 = 0.208, p = 0.023), phosphate (R^2 = - 0.170, p < 0.001) and sulfate (R^2 = 0.040, p = 0.003).
