# Supplementary material for: Nectar sugars and amino acids in day- and night-flowering Nicotiana species are more strongly shaped by pollinators’ preferences than organic acids and inorganic ions
Source: PLoS One. 2017 May 3;12(5):e0176865. doi: 10.1371/journal.pone.0176865 (PMC5415175; doi:10.1371/journal.pone.0176865)
Supplement: S5 Fig — (PDF) [file pone.0176865.s005.pdf]

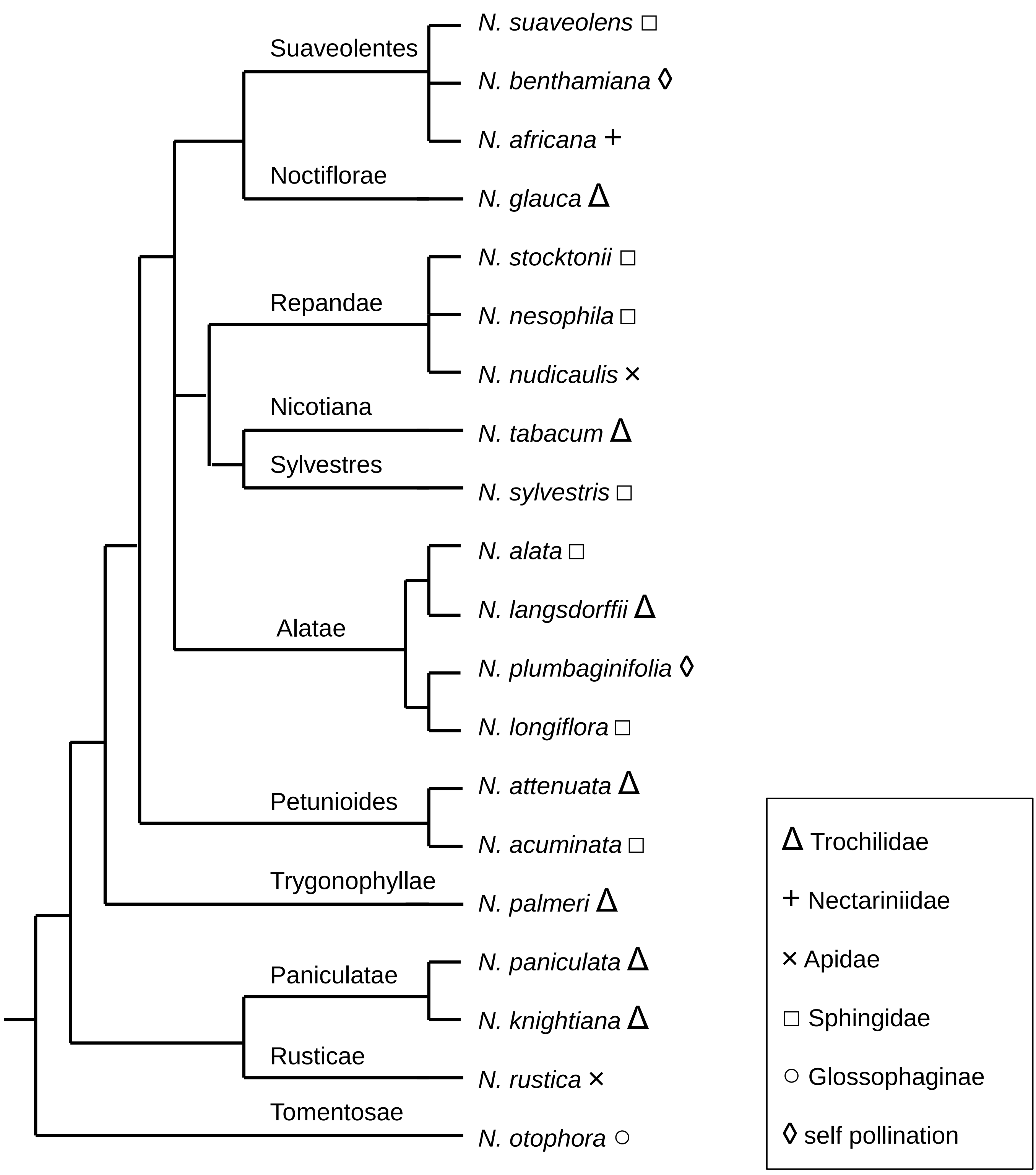

S5 Fig. Simplified phylogenetic tree of all examined *Nicotiana* species and their main pollinators

The symbols visualize the independence of pollinators from the sectional grouping (Goodspeed 1954, Knapp *et al*. 2004).
